# In Vitro Exposure to the Endocrine-Disrupting Chemical Climbazole Impairs Human Sperm Motility, Hormonal Signalling, and Mitochondrial Activity

**DOI:** 10.3390/cells14060427

**Published:** 2025-03-13

**Authors:** Eugenia Annunzi, Francesca Paola Luongo, Francesca Girolamo, Rosetta Ponchia, Sofia Passaponti, Paola Piomboni, Alice Luddi

**Affiliations:** 1 Department of Molecular and Developmental Medicine, University of Siena, 53100 Siena, Italy; 2 Department of Medicine, Surgery and Neuroscience, University of Siena, 53100 Siena, Italy

**Keywords:** endocrine-disrupting chemicals (EDCs), male fertility, climbazole (CBZ), semen quality, oxidative stress

## Abstract

This study explores the endocrine-disrupting effects of climbazole (CBZ), an environmental and lifestyle stressor, on male fertility. The impact of CBZ on sperm vitality, motility, and molecular pathways related to hormone receptors and apoptosis was evaluated, in non-capacitated and capacitated conditions. Gene expression of key components, including hormone receptors (*ESR1*, *ESR2*, *FSHR*, *AR*), apoptosis-related genes (*BAX*, *BCL2*), and *COX4l1* (involved in mitochondrial function), was analyzed. Protein tyrosine phosphorylation, a marker of capacitation, was also examined using immunofluorescence and Western blot analysis. We demonstrated that CBZ significantly reduced sperm vitality at concentrations above 25 µM and motility at 1 and 10 µM in non-capacitated and capacitated conditions. Changes in tyrosine phosphorylation patterns were also observed. Gene expression analysis revealed an upregulation of *ESR1*, *ESR2*, *FSHR*, and *BAX*, while *AR* and *COX4l1* expression were downregulated. These findings offer new insights into the potential endocrine-disrupting and cytotoxic effects of CBZ, highlighting its potential role in compromising male reproductive health.

## 1. Introduction

In recent decades, mounting evidence has underscored the detrimental effects of environmental and lifestyle stressors on human health, particularly through exposure to endocrine disruptors (EDCs). These chemicals, often found in pesticides, industrial products and pharmaceuticals, mimic or block the actions of natural hormones, leading to disruptions in physiological processes such as hormone synthesis, transport, metabolism, and action [1,2,3,4,5]. The most extensively studied mechanism of EDCs involves their binding to hormonal nuclear receptors, where they act as either agonists or antagonists, modifying the expression of hormone-responsive genes. Given the critical role of the endocrine system in regulating reproductive functions, the detrimental effects of EDCs on male reproductive health have raised significant concern. Indeed, these chemicals have been strongly associated with compromised semen quality, including decreased sperm count, reduced motility, and diminished vitality [6,7]. These impairments in sperm function can significantly reduce the chances of successful conception and pose long-term risks to reproductive health. However, understanding the impact of different EDCs on male fertility remains challenging, since these substances often act independently, synergistically, or in combination with other environmental stressors, complicating efforts to pinpoint their specific effects [8]. Moreover, while much research has focused on well-known EDCs, the impact of lesser-studied substances on male reproductive health remains underexplored. One such substance is climbazole (CBZ, CAS number: 38083-17-9), an antifungal agent commonly used in personal care products like shampoos and skincare treatments [9,10]. It works by inhibiting ergosterol, a key component of fungal cell membranes through suppression of cytochrome P450-dependent 14α-demethylase activity [11]. Beyond its antifungal effects, CBZ has been identified as an agonist of estrogen receptor α (ERα) and an antagonist of androgen receptor (AR), potentially disrupting hormonal regulation [12,13,14]. Additionally, CBZ affects enzymes involved in steroidogenesis, such as aromatase (CYP19), which is crucial for converting androgens into estrogens and maintaining sex hormone balance during development and in adulthood [15,16]. Furthermore, a recent study on adult zebrafish exposed to environmentally relevant concentrations of CBZ revealed significant alteration in glutathione and riboflavin metabolism, leading to oxidative stress and apoptosis [17]. Given its potential endocrine-disrupting effects, CBZ is currently under evaluation by the European Chemicals Agency (ECHA) for further assessment (https://echa.europa.eu/fr/substance-information/-/substanceinfo/100.048.870, accessed on 4 February 2025). Despite increasing concerns related to its health effects, there is a lack of direct evidence regarding CBZ’s potential as an endocrine disruptor on human male gametes.

This study aims to fill this gap in knowledge by investigating the in vitro effect of different doses of CBZ on sperm vitality and motility, while also exploring the biological mechanisms underlying its mode of action. Specifically, the study examines the expression of key hormone receptor pathways, including *ESR1*, *ESR2*, *FSHR*, and *AR*, as well as genes associated with apoptosis, such as *BAX* and *BCL-2*. The potential impact of CBZ on mitochondrial activity is also assessed by evaluating the expression of the mitochondrial marker *COX4l1* and the production of reactive oxygen species (ROS). Finally, the study explores if CBZ affects sperm capacitation—a crucial process for acquiring fertilization potential—by analyzing protein tyrosine phosphorylation—a key marker of sperm capacitation—and assessing its localization across distinct regions of the sperm membrane.

## 2. Materials and Methods

### 2.1. Participants

This study was conducted on a cohort of 20 males (age: 20–44 years) undergoing semen analysis for fertility evaluation at the Unit of Medically Assisted Reproduction, Siena University Hospital. The study protocol received approval from the Ethical Committee of the Siena University Hospital (approval ID CEAVSE, protocol number 18370, 2 October 2020); before participating, all subjects gave their written informed consent. A comprehensive clinical history was obtained for all participants, and subjects with possible preexisting causes of male infertility, such as varicocele, cryptorchidism, or endocrine disorders, were excluded. All individuals had a normal karyotype, and no history of chemotherapy, radiotherapy, or chronic illness.

### 2.2. Samples Collection and Semen Analysis

Semen samples were obtained by masturbation, after 3–5 days of sexual abstinence. After complete liquefaction of the sample at room temperature, the macroscopic and chemical–physical analysis was performed, evaluating parameters such as pH, volume, appearance, viscosity, and fluidization. The spermiogram was carried out according to WHO 2021 criteria [18]. Briefly, 10 µL of well-mixed semen were transferred to a Makler chamber and seminal parameters (sperm concentration, progressive sperm motility, non-progressive motility, morphology, vitality) were evaluated, and data reported are the mean value of the two observations. Moreover, sperm vitality was assessed using eosin dye. Based on these seminal parameters, normozoospermic subjects were selected for the study.

### 2.3. In Vitro Sperm Treatment with Climbazole

Climbazole, CAS number 38083-17-9, purity ≥ 98.0% (Supelco, St. Louis, MO, USA) was prepared in 0.1% DMSO to achieve final concentrations of 1, 5, 10, 25, and 50 µM. Semen samples were washed with Dulbecco’s phosphate-buffered saline without calcium and magnesium (DPBS, Corning, New York, NY, USA) and centrifuged at 600× *g* for 10 min. After centrifugation, the seminal plasma was separated from the pellet, which was resuspended in DPBS, and the sperm concentration was evaluated using a Mackler chamber, to prepare aliquots containing 40 million sperm cells. The aliquots were resuspended in either DPBS (non-capacitated sperm) or in capacitating medium (capacitated sperm). The capacitation medium consisted of 20 mM 4-(2-hydroxyethyl) piperazine-1-ethanesulfonic acid (HEPES), 112 mM NaCl, 3.1 mM KCl, 5 mM glucose, 21.7 mM sodium L-lactate, 1 mM sodium pyruvate, 0.3 mM Na_2_HPO_4_, 0.4 mM MgSO_4_ × 7H_2_O, 4.5 mM CaCl_2_ × 2H_2_O, 5 mg/mL BSA and 15 mM bicarbonate; pH = 7.4 ± 0.1; osmolality = 0.300 Osm/Kg ± 0.01. The prepared aliquots were distributed into control samples (vehicle, 0.1%DMSO) and CBZ-treated samples (at the described concentrations) under non-capacitating or capacitating conditions. Sperm variables, including vitality, motility, gene expression, and P-Tyr level, were analyzed after 0, and 90 min of incubation at 34 °C and 5% CO_2_.

### 2.4. Sperm Vitality and Motility Assessment

Sperm motility was measured according to the WHO (2021) guidelines [18]. Sperm vitality was determined using the eosin Y method. Briefly, a 10 µL aliquot was combined with 10 µL of eosin Y solution and analyzed under a light microscope at 400× magnification, adhering to WHO (2021) standards [18]. Dead sperm were pink colored, while live sperm cells remained colorless. This assessment was performed on all samples both before and following CBZ treatment.

### 2.5. Immunofluorescence and Acrosomal Staining

Immunofluorescence analysis was carried out following a previously published protocol [19]. Each sample was washed, smeared on a glass slide, and then fixed for 15 min in 4% paraformaldehyde. The cells were then permeabilized by 0.5% Triton X-100 in PBS for 5 min. Following this, blocking was performed with 5% bovine serum albumin (BSA)/1% normal goat serum (NGS) in PBS for 30 min. Spermatozoa were incubated overnight at 4 °C with the primary mouse anti-human P-Tyrosine antibody diluted in 1% NGS and 1% BSA in PBS. After three washes with PBS, the cells were incubated with the secondary antibody for 1 h at RT (see Supplementary Table S1). After an additional 3 washes with PBS, sperm were incubated for 30 min with 30 µg/mL fluorescein isothiocyanate-labeled Pisum Sativum agglutinin (FITC-PSA) to label the acrosome. After a 10 min PBS wash, nuclei were counterstained with DAPI, and slides were mounted with DABCO antifade solution. Sperm samples were analyzed using a Leica AF6500 Integrated System for Imaging and Analysis (Leica Microsystems, Wetzlar, Germany) equipped with the LAS AF software, version 2.7.3.9723. P-Tyr staining patterns were classified according to published criteria [20], and the percentages of acrosome-reacted and intact acrosome sperm were determined.

### 2.6. Western Blot Analysis

Western blotting was performed as previously described [21]. Briefly, 50 μg of total proteins were denatured, separated on 8% polyacrylamide gel, and then transferred to a nitrocellulose membrane (GE Healthcare, Chicago, IL, USA). After blocking for 1 h in 5% non-fat dry milk, the membrane was incubated overnight at 4 °C with primary antibodies (see Supplementary Table S1), diluted in 1% non-fat dry milk/TTBS (TBS containing 0.2% Tween 20). After TTBS washing, the membrane was incubated with the appropriate horseradish peroxidase (HRP)-conjugated secondary antibody (see Supplementary Table S1). Immunostained bands were visualized using chemiluminescence with ImageQuant LAS 4000 (GE Healthcare) and band densitometry was quantified using Image J, version 1.54k (https://imagej.net/ij/, accessed on 4 February 2025).

### 2.7. RNA Extraction, Retrotranscription and qRT-PCR

The total RNA was extracted using a QIAzol lysis reagent (QIAGEN, Germantown, MD, USA), according to a previously published protocol [22]. Briefly, 1 mL of QIAzol and 200 µL of chloroform were added to each sample. The mixture was centrifuged at 12,000 rcf for 15 min at 4 °C. The aqueous phase was transferred to a new tube, and an equal volume of cold isopropanol was added. After incubating for 30 min at 4 °C, RNA was pelleted by centrifugation at 12,000 rcf for 15 min at 4 °C. After two washes with 75% ethanol, the pellet was air-dried and resuspended in nuclease-free water. RNA integrity was assessed using a Thermo Scientific™ NanoDrop™ One/OneC Microvolume UV-Vis Spectrophotometer (Thermo Fisher Scientific, Waltham, MA, USA). For cDNA synthesis, 500 ng of RNA was reverse transcribed using the SuperScript IV VILO Master Mix (Invitrogen, Carlsbad, CA, USA) according to the manufacturer’s instructions. The resulting cDNA was then used for quantitative real-time PCR (qRT-PCR) with the CFX Connect Real-Time PCR System (Bio-Rad Laboratories, Hercules, CA, USA) and specific primer pairs (Supplementary Table S2). Each sample was processed in triplicate in a volume of 10 µL, of which 5 µL consisted of the PowerTrack™ SYBR Green Master Mix for qPCR (Applied Biosystems, Waltham, MA, USA), to which 1 µM of each primer and 3 µL of nuclease-free water were added. The program used was as follows: 95 °C for 10 min followed by 45 cycles composed of 95 °C for 3 s, the specific pairing temperature for each primer for seconds. To determine the most stable reference genes, we used RefFinder-master (https://www.ciidirsinaloa.com.mx/RefFinder-master/?type=reference, accessed on 4 February 2025), identifying *GAPDH* as the most stable, followed by *B2M*, and *18S* as the least stable; thus, gene expression analyses were normalized using *GAPDH* and *B2M* (Supplementary Figure S1). The results obtained were analyzed by applying a comparative method (∆∆Ct). According to this method, quantities are expressed relative to a calibrator sample representing the unit quantity of the gene of interest and all other samples are expressed as n times the calibrator.

### 2.8. ROS Detection

2′,7′-dichlorodihydrofluorescein diacetate dye (DCFH-DA) (Life Technologies Corp., Carlsbad, CA, USA) was used to detect intracellular reactive oxygen species (ROS). Briefly, samples were incubated with DCFH-DA solution (10 μM in PBS) at 37 °C for 15 min. Then, the cells were centrifuged, and the supernatant was collected. Fluorescence was measured using excitation and emission wavelengths of 480 and 520 nm, respectively, using Synergy HTX multi-mode reader (BioTek, Winooski, VT, USA) and normalized to the number of sperm cells.

### 2.9. JC-1 for Assessing Mitochondrial Membrane Potential (MMP)

Alterations in MMP of spermatozoa under non-capacitating and capacitating conditions treated with 1 and 10 µM of CBZ, were evaluated using the cation 5,5′,6,6′-tetrachloro-1,1′,3,3′-tetraethylbenzimidazolcarbocyanine iodide (JC-1) dye (Molecular Probes, Eugene, OR, USA). Briefly, SPZ were incubated in the dark with 1 μg/mL JC-1 dye diluted in PBS for 20 min at 37 °C. MMP levels were assessed using a Leitz Aristoplan fluorescence microscope (Leica, Wetzlar, Germany) with a 490 nm excitation light and a 530 nm barrier filter. A minimum of 300 sperm were analyzed at 630× magnification. Sperm with high MMP exhibited red fluorescence at the midpiece due to JC-1 aggregate formation, while those with low MMP displayed a diffuse green signal, indicating JC-1 in its monomeric form. Results are reported as a percentage red/green ratio.

### 2.10. DNA Fragmentation Analysis

The microscopic acridine orange test was performed following established protocols [23,24] to assess sperm DNA integrity. Three smears per sample were prepared on glass slides, air-dried, and fixed in freshly prepared Carnoy’s solution (methanol/glacial acetic acid, 3:1) for at least three hours or overnight. After air-drying, slides were incubated in a buffer (80 mmol/L citric acid, 15 mmol/L Na_2_HPO_4_, pH 2.5) at 75 °C for five min to assess chromatin stability. Slides were then stained for five min in freshly prepared acridine orange (AO) solution (2.5 mL of 1% AO stock, 10 mL 0.1 mol/L citric acid, 400 µL 0.3 mol/L Na_2_HPO_4_·H_2_O) or with AO at 0.2 mg/mL. After washing, slides were wet-mounted and analyzed using a Leica AF6500 Integrated System for Imaging and Analysis (Leica Microsystems, Wetzlar, Germany) equipped with the LAS software. Sperm with intact DNA fluoresced green, while those with DNA damage showed yellow-green to red fluorescence. The percentage of intact chromatin was calculated by counting 100–200 sperm per slide. Two independent observers evaluated each slide, and the mean value was used for analysis. The percentage of apoptotic spermatozoa was assessed using the TUNEL assay (In Situ Cell Death Detection Kit, Roche, Basel, Switzerland). Sperm smears were fixed with 4% paraformaldehyde in PBS for 15 min, washed, and treated with 0.3% H_2_O_2_ in methanol for 1 h to quench peroxidase activity. Permeabilization was performed with 0.1% Triton X-100 at 4 °C for 5 min. Slides were incubated with TUNEL reaction mixture (50 μL) at 37 °C for 1 h, followed by converter-POD (50 μL) and DAB staining for color development. After dehydration, clearing, and mounting, samples were analyzed under a fluorescence microscope (100× magnification), counting 200 sperm nuclei per sample. For negative controls, the TUNEL reaction mixture was replaced with a label solution. Spermatozoa were classified as TUNEL− (light green, non-apoptotic) or TUNEL+ (bright green, apoptotic).

### 2.11. Statistical Analysis

Results were presented as mean ± Standard Deviation (SD). Data results were determined by two-way ANOVA, followed by multiple comparisons or Bonferroni’s post hoc tests between control and treated samples for the calculation of statistical significance. For the analysis of the DCF percentage, we used one-way ANOVA with Dunn’s multiple comparison test. Analysis of the results was performed using GraphPad Prism 8 for Windows (v.8.0.2. Dotmatics, Boston, MA, USA), and results with *p* < 0.05 were considered significant.

## 3. Results

### 3.1. CBZ Affects Sperm Vitality and Motility

**Figure 1:**
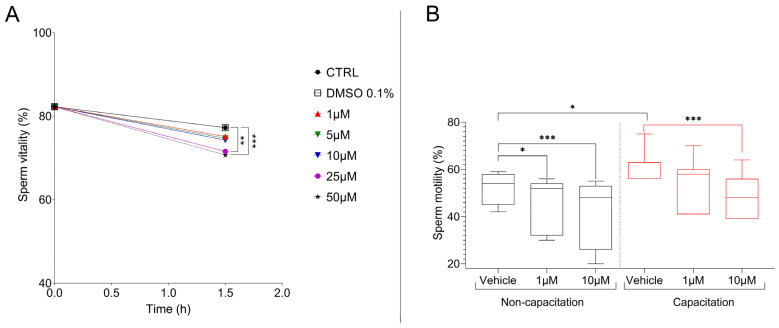
(**A**) Percentage of sperm vitality in relation to the time between control, vehicle (DMSO 0.1%) and treatment with CBZ. (**B**) Percentage of sperm motility in relation to the time between vehicle and treatment with CBZ. Significant differences are indicated with Bonferroni correction * *p* < 0.05; ** *p* < 0.01; *** *p* < 0.001.

The seminal parameters of the samples included in this study are summarized in Table 1, with all samples meeting the WHO 2021 criteria for normozoospermia. To evaluate the effect of CBZ, ejaculated sperm samples were treated in vitro with increasing concentrations of CBZ and its effect on sperm vitality was assessed. No significant differences in vitality were observed between the untreated samples (control group) and 0.1% DMSO-treated sperm (vehicle group) (*p* > 0.05). Similarly, samples treated with CBZ at concentrations of 1, 5 and 10 µM showed no significant reduction in vitality compared to the control and vehicle groups after 90 min of incubation. However, a significant decrease in vitality was observed in samples exposed to 25 (*p* = 0.0012) and 50 µM (*p* ≤ 0.0001) of CBZ relative to the control and vehicle groups (Figure 1A and Supplementary Table S3). Based on these findings, subsequent analyses were conducted using only the vehicle (0.1% DMSO) and the highest non-cytotoxic doses of CBZ (1 and 10 µM). To evaluate whether CBZ treatment affects sperm motility, treatments were performed under both non-capacitation condition and in vitro capacitation. Following the treatments, a significant reduction in motility was observed under non-capacitation conditions in samples treated with CBZ compared to the vehicle group (1 µM *p* = 0.0285; 10 µM *p*= 0.0002). Instead, in capacitated samples, this decrease was detected only in cells treated with 10 µM of CBZ (*p* = 0.0005) (Figure 1B and Supplementary Table S4).

### 3.2. Effects of CBZ on Sperm Capacitation

**Figure 2:**
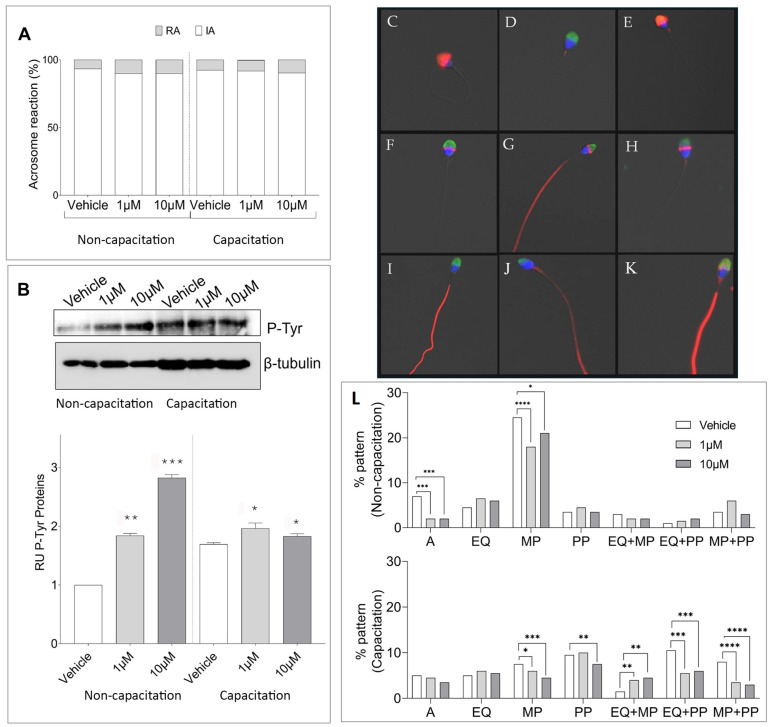
(**A**) Percentage of Acrosome reaction staining in non-capacitation and capacitation conditions. ((**B**)-upper part) Representative Western blot analysis of P-Tyr in human sperm exposed to Vehicle, 1 and 10 µM of β-tubulin was used as a loading control. ((**B**)-lower part) Bar graph showing the overall relative intensity of the bands, quantified by computer-assisted densitometric analysis. (**B**) is representative of 3 independent experiments (**C**–**K**). Representative Immunofluorescent images illustrating P-Tyr localization (red) and PSA staining (green) in human sperm, with nuclei counterstained in blue. (**C**) acrosome, A; (**D**) Mid piece, MP; (**E**) Acrosome and Mid piece, A+MP; (**F**) Equatorial region, EQ; (**G**) Equatorial region and Principal Piece, EQ+PP; (**H**) Equatorial region and Mid Piece, EQ+MP; (**I**) Principal Piece, PP; (**J**) Equatorial segment and Principal Piece, EQ+PP; (**K**) Acrosome and Principal Piece, A+PP, Magnification 6300×. (**L**) Quantification of P-Tyr staining patterns based on subcellular localization in sperm under non-capacitation and capacitation conditions. Data represent the mean proportion of each pattern (±SD). A total of 200 sperm were analyzed per sample. * *p* < 0.05; ** *p* < 0.01; *** *p* < 0.001; **** *p* < 0.0001.

We further investigated the impact of CBZ on the acrosome reaction, a crucial process regulated by physiological and molecular changes during sperm capacitation that enables sperm to penetrate the oocyte zona pellucida [25]. Assessing acrosome integrity provides valuable insights into CBZ’s potential effects on human sperm function. As illustrated in Figure 2A, CBZ treatment in non-capacitated sperm resulted in a higher percentage of acrosome-reacted cells, though this increase did not reach statistical significance. Moreover, a chi-square test of independence was conducted to assess whether CBZ exposure affected acrosome integrity in both non-capacitated and capacitated sperm, but no significant association was found between these variables. We, therefore, measured the tyrosine phosphorylation (P-Tyr) level, a well-established marker of sperm capacitation progression [26] in CBZ-treated sperm. The results of the Western blot analysis comparing Tyr-P protein levels across the study groups are presented in Figure 2B. A prominent band at approximately 200 kDa, corresponding to a family of tyrosine-phosphorylated proteins was observed in all samples. Quantitative analysis of these bands, shown in Figure 2B, confirmed the well-known increase in P-Tyr levels during capacitation, which was further amplified in CBZ-treated samples at both 1 and 10 µM. Interestingly, the increase in P-Tyr levels was more pronounced in CBZ-treated sperm under non-capacitating conditions.

To provide deeper insights, we also analyzed the distribution of P-Tyr in response to the treatment with CBZ in both non-capacitated and capacitated sperm. Phosphorylated tyrosine residues were detected in the following regions: the acrosomal cap, the equatorial segment, the midpiece, the principal piece, and various combinations of these regions (Figure 2C–K). Examined patterns of P-Tyr expression revealed treatment-related alterations, as reflected by their varied percentages. Notably, in non-capacitated sperm, P-Tyr residues showed a significant decrease exclusively in the acrosomal and midpiece compartments of CBZ-treated sperm compared to the vehicle (Figure 2L). Under capacitating conditions, CBZ-treated sperm exhibited significant modulation of P-Tyr staining in the tail, specifically in the midpiece and principal piece, including combinations with the equatorial segment and acrosome (Figure 2L).

### 3.3. CBZ Treatment Modulates Hormone Receptor Expression in Sperm

To further explore CBZ’s endocrine-disrupting potential, we examined its impact on key hormone receptor expression, in both non-capacitated and capacitated sperm.

**Figure 3:**
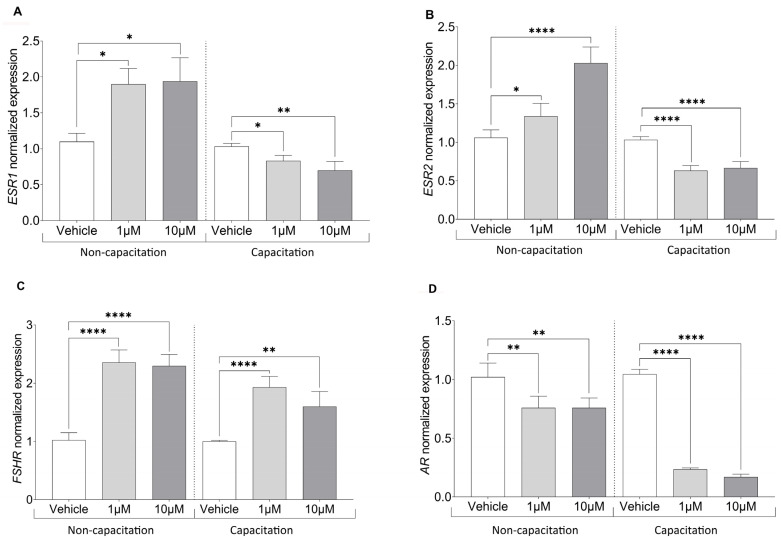
Normalized gene expression of hormone receptors in sperm treated with CBZ calculated by Delta–Delta Ct (ΔΔCt) method. (**A**) Estrogen Receptor 1 (*ESR1*), (**B**) Estrogen Receptor 2 (*ESR2*), (**C**) Follicle Stimulating Hormone Receptor (*FSHR*) and (**D**) Androgen Receptor (*AR*). Significant differences are indicated by Bonferroni-corrected *p* value: * *p* < 0.05; ** *p* < 0.01; **** *p* < 0.0001.

In non-capacitated samples, *ESR1* and *ESR2* levels significantly increased following treatment with 1 µM CBZ (*p* = 0.0189 and *p* = 0.0350, respectively) and 10 µM CBZ (*p* = 0.0129 and *p* ≤ 0.0001, respectively). By contrast, in capacitated sperm treated with CBZ, we observed a significant decrease in the expression of *ESR1*, *ESR2* genes when compared to the vehicle (Figure 3A,B; for *p*-value Bonferroni adjustment see Supplementary Table S5). Additionally, *FSHR* expression was significantly higher in CBZ-treated sperm at both 1 µM and 10 µM concentrations (*p* ≤ 0.0001), regardless of capacitation status. In contrast, *AR* gene expression was significantly reduced in samples treated with 1 (*p* = 0.0015) and 10 µM (*p* = 0.0015) CBZ compared to the vehicle, under both non-capacitating and capacitating conditions (Figure 3C,D).

**Figure 4:**
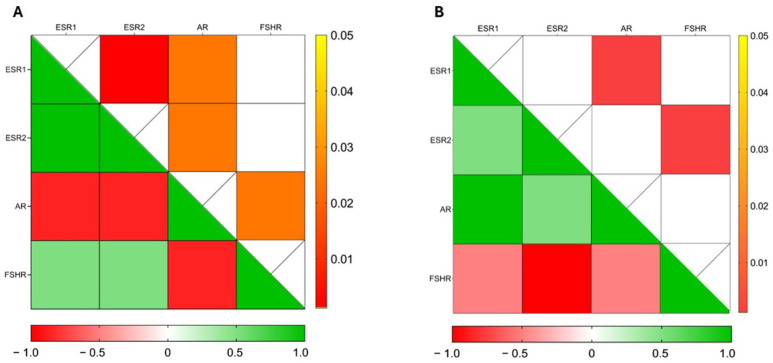
Heat maps illustrate the correlation analysis between gene expression of *ESR1*, *ESR2*, *AR* and *FSHR* in (**A**) non-capacitated and (**B**) capacitated sperm. Cells filled in green to red gradient of the heat maps (lower part) represent Spearman’s r; cells filled in yellow to red gradient (upper part) represent *p* values (empty cells stand for *p* values greater than 0.05).

Spearman’s correlation analyses showed a significant positive correlation between *ESR1* and *ESR2* expression (r = 1.0; *p* = 0.001) in non-capacitated sperm, while both genes exhibited a significant negative correlation with *AR* (r = −0.8660; *p* = 0.024) (Figure 4A). Conversely, under capacitated conditions, a significant positive correlation was observed only between *ESR1* and *AR* (r = 1.0; *p* = 0.001) (Figure 4B).

### 3.4. Climbazole Upregulates Pro-Apoptotic Gene Expression in Human Sperm

**Figure 5:**
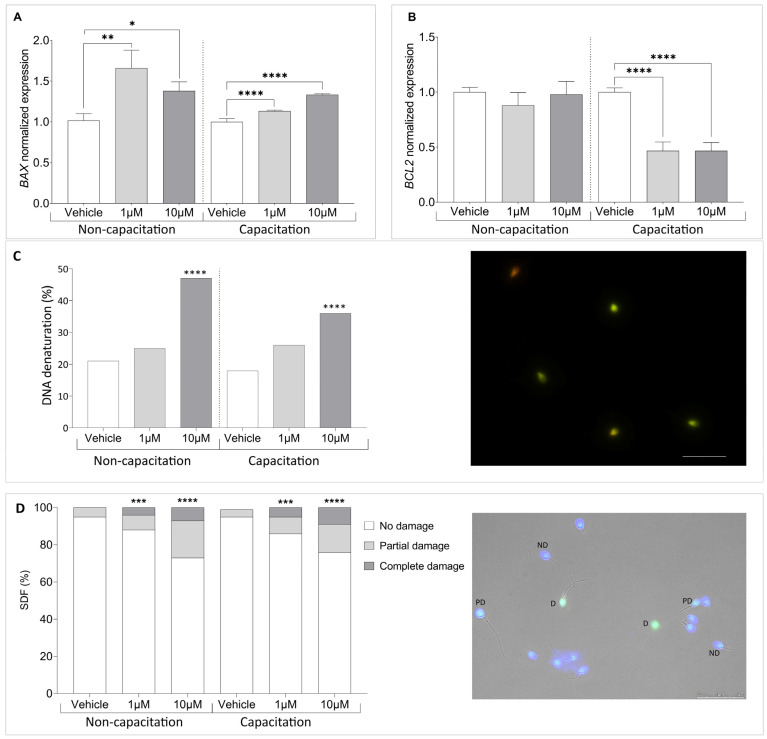
Normalized gene expression of (**A**) *BAX* and (**B**) *BCL2* in vehicle-treated versus CBZ-treated sperm, calculated using Delta–Delta Ct (ΔΔCt) method. (**C**) Left panel: Percentage of DNA denaturation. Right panel: Representative image of Acridin orange staining, showing sperm with normally condensed, non-fragmented (green) and decondensed or damaged chromatin (red). (**D**) TUNEL assay. Sperm DNA fragmentation index (SDF) percentage categorizing sperm with no damage (ND), partial damage (PD) or complete DNA damage (D). Scale bar: 25 µm. Significant differences are indicated by Bonferroni-corrected *p* value: * *p* < 0.05; ** *p* < 0.01; *** *p* < 0.001; **** *p* < 0.0001.

Building on evidence that CBZ modulates apoptotic pathways by affecting key apoptotic gene expression [17,27], we analyzed the expression of apoptosis-regulating genes *BAX* and *BCL2* in sperm exposed to CBZ for 90 min. As illustrated in Figure 5A, the mRNA level of the pro-apoptotic gene *BAX* significantly increased in non-capacitated sperm treated with 1 µM (*p* = 0.0072) and 10 µM (*p* = 0.0281) CBZ compared to the vehicle. A similar pattern was observed in capacitated sperm, where *BAX* mRNA levels were significantly higher in sperm treated with 1 (*p* ≤ 0.0001) and 10 µM (*p* ≤ 0.0001) CBZ compared to the vehicle. Interestingly, while the expression of the anti-apoptotic gene *BCL2* remained unchanged in non-capacitated sperm, a significant reduction in *BCL2* expression was detected in capacitated sperm treated with CBZ (1 µM *p* ≤ 0.0001; 10 µM *p* ≤ 0.0001) (Figure 5B).

Based on this finding, we investigated sperm DNA status. First, we assessed chromatin denaturation and integrity using acridine orange staining. As shown in Figure 5C, exposure to 10 µM CBZ resulted in a significant decrease in DNA condensation under both non-capacitation (*p* < 0.0001) and capacitation (*p* < 0.0001) conditions. To further evaluate sperm DNA integrity, we performed a TUNEL assay, a direct method for detecting DNA strand breaks. Unlike acridine orange staining, which assesses chromatin denaturation, the TUNEL assay provides a more precise evaluation of apoptosis-related sperm DNA damage. As shown in Figure 5D, we observed an increase in DNA damage in samples treated with both 1 µM (*p* < 0.001) and 10 µM (*p* < 0.0001) compared to the vehicle, in both non-capacitation and capacitation conditions.

### 3.5. Climbazole Alters Mitochondrial Function in Human Sperm

**Figure 6:**
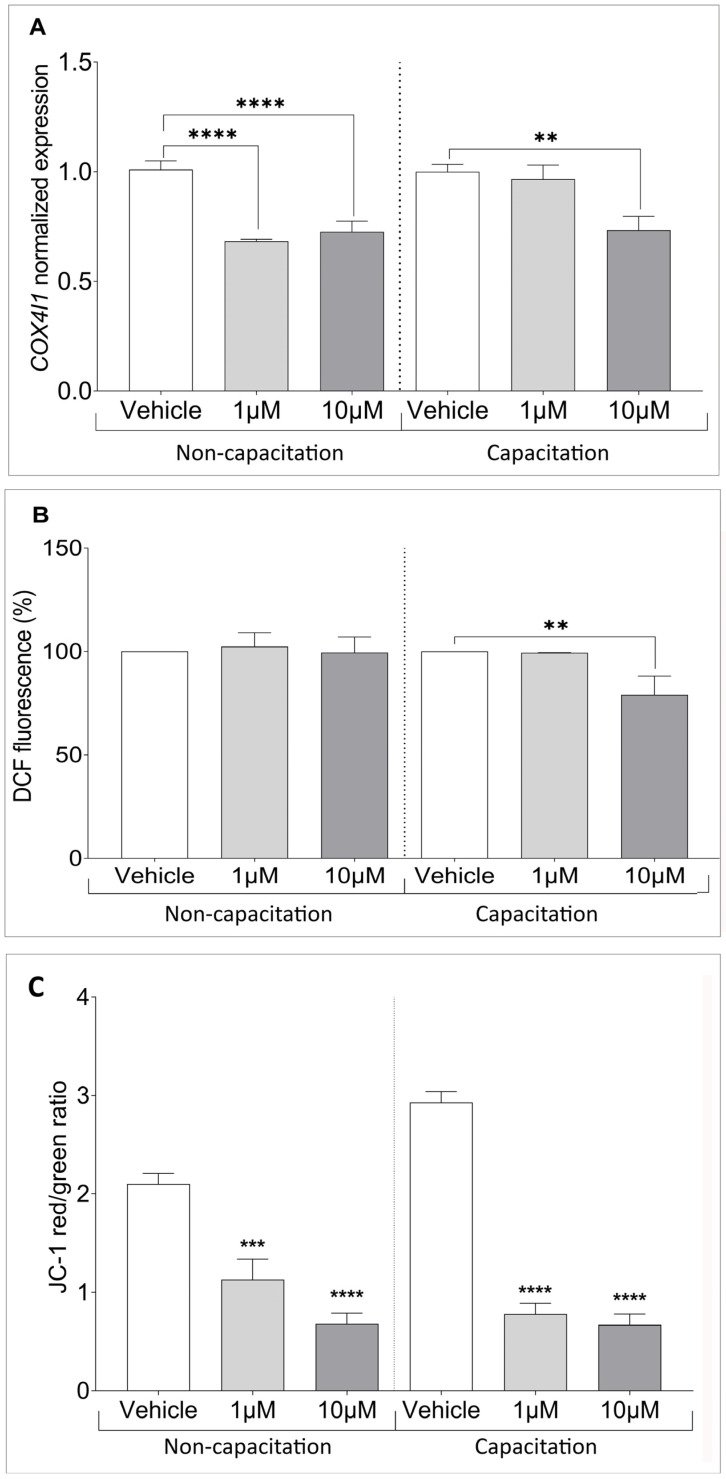
(**A**) Normalized gene expression of *COX4I1* between vehicle-treated and CBZ-treated samples, calculated by Delta–Delta Ct (ΔΔCt) method. (**B**) Percentage of Dichlorofluorescein (DCFH-DA) in relation to the time between vehicle and treatment with CBZ. (**C**) Bar plot showing JC-1 staining with red (high MMP)/green (low MMP) ratio. Significant differences are indicated with Bonferroni-corrected *p* value: ** *p* < 0.01; *** *p* < 0.001; **** *p* < 0.0001.

To further explore the mechanisms underlying CBZ’s effects, we focused on mitochondria as a potential target. This approach is supported by evidence from the literature indicating that CBZ can impair mitochondrial function and structure, as well as induce oxidative stress [28]. Given their pivotal role in energy metabolism and their essential contribution for the acquisition of sperm fertilizing capacity, mitochondria are a critical focus for understanding CBZ’s impact on male reproductive health. As a first step, we examined the expression of *COX4l1*, a critical gene involved in mitochondrial function and energy production [27,29]. Our results revealed a significant reduction in *COX4l1* expression in non-capacitated sperm treated with CBZ, with decreases observed at both 1 (*p* < 0.0001) and 10 µM (*p* < 0.0001) compared to the vehicle. In capacitated sperm, however, a significant decrease was only observed at the highest CBZ dose of 10 µM (*p* = 0.0024) compared to the vehicle (Figure 6A). A reduction in COX4l1 activity can lead to electron leakage within the mitochondrial electron transport chain, potentially increasing ROS production. To further elucidate the mechanism underlying CBZ’s effects on spermatozoa, we assessed intracellular ROS levels using DCF staining. While no significant differences were observed in non-capacitated sperm, under capacitating conditions, a significant decrease in ROS levels was detected in spermatozoa treated with 10 µM of CBZ compared to the vehicle (Figure 6B).

To further assess mitochondrial activity, we analyzed the altered state of MMP through the red/green fluorescence emission ratio of JC-1 in the mid piece of SPZ. Our results showed a decrease in the ratio and, consequently, an increase in green fluorescence, indicating a low MMP in samples treated with 1 µM (*p* < 0.001) and 10 µM (*p* < 0.0001) compared to the vehicle, in both non-capacitation and capacitation conditions (Figure 6C).

## 4. Discussion

Our study examined the effects of climbazole, a chemical commonly found in dandruff treatment products, on human spermatozoa, revealing a detrimental impact on sperm vitality and motility, changes in hormone receptor expression, and impairment of mitochondrial function. To the best of our knowledge, no prior studies have explored the effects of this compound on human male reproduction. The only relevant study examining CBZ’s impact demonstrated in vivo effects on zebrafish testes, where CBZ disrupted the synthesis of essential sex hormones and reduced sperm production [28]. These findings, combined with the endocrine-disrupting properties previously reported for CBZ, underscore the importance of understanding its potential reproductive toxicity in humans. Unlike other studies conducted on mammalian cellular models, where CBZ prolonged exposure, up to eight days, was settled at a concentration ranging from 0.1 to 10 µM [13,30,31], our in vitro sperm model requires a shorter exposure time due to the unique nature of sperm cells and the challenges in maintaining their viability and functionality in vitro. Despite these limitations, the concentrations used in our study are comparable to those in previous research [13,30], enabling meaningful comparisons while offering insight into acute effects. This distinction allows for a closer alignment with physiological constraints, making our findings particularly relevant to male reproductive toxicology. CBZ concentrations up to 10 µM did not significantly compromise sperm viability after 90 min of incubation, suggesting that membrane integrity remains unaffected at lower doses. However, higher concentrations (25 and 50 µM) significantly reduced sperm viability, reinforcing concerns about its cytotoxicity at elevated doses [32]. At lower, non-lethal concentrations (1 and 10 µM) the CBZ impaired sperm motility, with significant reductions observed in non-capacitated sperm at both concentrations. In contrast, under capacitating conditions, only the higher concentration of 10 µM resulted in decreased motility. This differential response highlights the compound’s potential to interfere with signaling pathways critical for capacitation, which is essential for enhanced motility and fertilization capability. The analysis of tyrosine phosphorylation further supports this disruption. CBZ significantly altered this process, particularly affecting the localization of phosphorylated residues. Tyrosine phosphorylation is a key regulatory mechanism that modifies protein conformation, affecting sperm signaling pathways and motility through AKAPs, PKAs, and glycolytic enzymes [20]. CBZ significantly enhanced this process even in non-capacitation conditions, suggesting that this EDC may induce premature capacitation, potentially compromising sperm reproductive efficiency within the female reproductive tract. Moreover, CBZ affects the localization of phosphorylated residues. Under non-capacitation conditions, reductions were observed in the acrosome and midpiece regions, suggesting early interference with activation signals. In capacitated sperm, the alterations were more pronounced, affecting all tail and the equatorial region compartments and disrupting combinations of phosphotyrosine localization critical for motility regulation and acrosomal exocytosis. These findings further support the notion that CBZ may accelerate capacitation, which is characterized by tail phosphorylation [33]. Interestingly, Western blot analysis revealed an overall increase in tyrosine phosphorylation following CBZ treatment under both non-capacitated and capacitated conditions, with the strongest effects at 1 µM. This paradoxical increase may reflect a compensatory response to disrupted signaling, resulting in dysregulated capacitation. CBZ’s impact extended to the expression of genes involved in hormonal signaling and apoptosis, with condition-specific effects observed. In non-capacitated sperm, CBZ upregulated *ESR1*, *ESR2*, and *FSHR*, reflecting potential estrogenic or gonadotropin-mimicking effects, while downregulating *AR*, suggesting an androgen-antagonist mechanism. These findings are consistent with previous reports of CBZ’s endocrine-disrupting properties, including its estrogen agonist and androgen antagonist actions observed in mammalian cell models [12,13,14]. Under capacitating conditions, however, *ESR1* and *ESR2* expression were downregulated, while *FSHR* expression remained elevated, indicating that CBZ exerts distinct effects depending on the physiological state of the sperm. The expression of apoptotic markers further highlighted CBZ’s potential reproductive toxicity. The pro-apoptotic gene *BAX* was consistently upregulated in both non-capacitation and capacitation conditions, while the anti-apoptotic gene *BCL2* was significantly downregulated in capacitated sperm. These alterations indicate an activation of apoptotic pathways that may contribute to reduced viability and motility. Notably, *BAX* expression negatively correlated with both motility and vitality, emphasizing its role in CBZ-induced sperm dysfunction. Notably, we confirmed that DNA damage and fragmentation increased upon treatment in a dose-dependent manner, definitively demonstrating that CBZ not only can induce cell death at higher doses, but also exerts a detrimental transgenerational effect. Additionally, the downregulation of *COX4l1*, a gene associated with mitochondrial function [34], suggests mitochondrial dysfunction as a key factor in CBZ-induced sperm quality impairment. While oxidative stress is a well-known mediator of sperm dysfunction and male infertility [35], our findings showed that CBZ treatment at 10 µM under capacitating conditions resulted in a significant reduction in ROS presence. This contrasts with the typical effect of EDCs, which often increase ROS [6,36,37] levels, suggesting that CBZ may reduce oxidative stress through impaired mitochondrial function rather than enhancing oxidative balance. The downregulation of *COX4l1* and the associated reduction in mitochondrial activity could, therefore, contribute to the decline in sperm motility observed under capacitating conditions. This finding aligns with the significant decrease in MMP, suggesting mitochondrial dysfunction that may impair ATP production and ultimately reduce sperm motility. This finding underscores the complexity of CBZ’s effects on sperm, where mitochondrial dysfunction and oxidative stress may act in tandem to impair sperm function.

## 5. Conclusions

Our findings demonstrate that CBZ significantly impairs sperm functionality, even at sub-lethal concentrations, by disrupting hormonal signaling and dysregulating tyrosine phosphorylation. These effects compromise critical processes such as capacitation, which occurs within the female reproductive tract and is essential for motility enhancement and fertilization. Moreover, the observed induction of apoptosis by CBZ raises serious concerns about its impact on sperm DNA integrity. As gametes are responsible for delivering intact genetic material to the oocyte, DNA damage caused by strand breaks could hinder embryonic development. The potential contamination of the female reproductive tract with CBZ further underscores its capacity to reduce fertilization potential in vivo.

Future studies should focus on unraveling the molecular pathways through which CBZ disrupts these essential processes, including its interactions with hormonal receptors, mitochondrial function, and apoptotic pathways. Additionally, exploring the long-term and transgenerational consequences of CBZ exposure will be vital to comprehensively assessing its impact on reproductive health and fertility.

## Figures and Tables

**Table 1:** Demographic characteristics of participants.

Semen Parameters	Mean (SD)
Volume (mL)	3.28 ± 1.71
pH	7.50 ± 0.16
Sperm Concentration (10^6^/mL)	79.50 ± 36.90
Total sperm number (10^6^)	217.1 ± 81.12
Motility (%)	61.17 ± 3.67
Vitality (%)	83.50 ± 2.48
Morphology (%)	8.16 ± 2.71

## Data Availability

All data underlying the study are available from the corresponding authors.

## Supplementary Materials

The following supporting information can be downloaded at: https://www.mdpi.com/article/10.3390/cells14060427/s1, Table S1: Characteristics of primary and secondary antibodies used for immunofluorescence staining and Western Blots. Table S2: List of primers used for qRT–PC. Figure S1: Gene stability analysis between housekeeping genes: *GAPDH*, *B2M*, *18S.* Table S3: Percentage of sperm vitality in relation to time between control, DMSO 0.1% and treatment with CBZ. Table S4: Percentage of sperm motility in relation to time between vehicle and treatment with CBZ. Table S5: Normalized gene expression between vehicle and samples treated with CBZ calculated by Delta–Delta Ct (ΔΔCt) method. Table S6: Spearman’s r and *p*-value from the correlation analysis between *ESR1*, *ESR2*, *AR*, and *FSHR* reported through the heatmap graph.

## References

[1] Jenardhanan P., Panneerselvam M., Mathur P.P. (2016). Effect of Environmental Contaminants on Spermatogenesis. Semin. Cell Dev. Biol..

[2] Brehm E., Flaws J.A. (2019). Transgenerational Effects of Endocrine-Disrupting Chemicals on Male and Female Reproduction. Endocrinology.

[3] Luongo F.P., Annunzi E., Girolamo F., Belmonte G., Ponchia R., Piomboni P., Luddi A. (2024). Assessment of Sperm Chromosomal Abnormalities Using Fluorescence in Situ Hybridization (FISH): Implications for Reproductive Potential. J. Assist. Reprod. Genet..

[4] Gore A.C., Chappell V.A., Fenton S.E., Flaws J.A., Nadal A., Prins G.S., Toppari J., Zoeller R.T. (2015). EDC-2: The Endocrine Society’s Second Scientific Statement on Endocrine-Disrupting Chemicals. Endocr. Rev..

[5] Bergman Å., Heindel J.J., Jobling S., Kidd K.A., Zoeller R.T. (2012). State of the Science of Endocrine Disrupting Chemicals—2012: An Assessment of the State of the Science of Endocrine Disruptors Prepared by a Group of Experts for the United Nations Environment Programme (UNEP) and WHO.

[6] Virant-Klun I., Imamovic-Kumalic S., Pinter B. (2022). From Oxidative Stress to Male Infertility: Review of the Associations of Endocrine-Disrupting Chemicals (Bisphenols, Phthalates, and Parabens) with Human Semen Quality. Antioxidants.

[7] Evans T.J. (2015). Reproductive Toxicity and Endocrine Disruption of Potential Chemical Warfare Agents. Handbook of Toxicology of Chemical Warfare Agents.

[8] Axelsson J., Rylander L., Rignell-Hydbom A., Lindh C.H., Jönsson B.A.G., Giwercman A. (2015). Prenatal Phthalate Exposure and Reproductive Function in Young Men. Environ. Res..

[9] Turner G.A., Matheson J.R., Li G.-Z., Fei X.-Q., Zhu D., Baines F.L. (2013). Enhanced Efficacy and Sensory Properties of an Anti-dandruff Shampoo Containing Zinc Pyrithione and Climbazole. Int. J. Cosmet. Sci..

[10] Wood A.J.J., Como J.A., Dismukes W.E. (1994). Oral Azole Drugs as Systemic Antifungal Therapy. N. Engl. J. Med..

[11] Zarn J.A., Brüschweiler B.J., Schlatter J.R. (2003). Azole Fungicides Affect Mammalian Steroidogenesis by Inhibiting Sterol 14 Alpha-Demethylase and Aromatase. Environ. Health Perspect..

[12] Kenda M., Karas Kuželički N., Iida M., Kojima H., Sollner Dolenc M. (2020). Triclocarban, Triclosan, Bromochlorophene, Chlorophene, and Climbazole Effects on Nuclear Receptors: An in Silico and in Vitro Study. Environ. Health Perspect..

[13] Ndiaye D., Perceau M., Lorcin M., Denis F., Gaté L. (2024). Antifungal Climbazole Alters Androgenic Pathways in Mammalian Cells. Toxicol. Vitr..

[14] Zhang H., Chen Z., Qi Z., Yan S., Wei W., Liu G., Cai Z. (2019). Analysis of Transcriptional Response in Zebrafish Eleutheroembryos Exposed to Climbazole: Signaling Pathways and Potential Biomarkers. Environ. Toxic. Chem..

[15] Trösken E.R., Fischer K., Völkel W., Lutz W.K. (2006). Inhibition of Human CYP19 by Azoles Used as Antifungal Agents and Aromatase Inhibitors, Using a New LC–MS/MS Method for the Analysis of Estradiol Product Formation. Toxicology.

[16] Blakemore J., Naftolin F. (2016). Aromatase: Contributions to Physiology and Disease in Women and Men. Physiology.

[17] Zou T., Liang Y.-Q., Liao X., Chen X.-F., Wang T., Song Y., Lin Z.-C., Qi Z., Chen Z.-F., Cai Z. (2021). Metabolomics Reveals the Reproductive Abnormality in Female Zebrafish Exposed to Environmentally Relevant Levels of Climbazole. Environ. Pollut..

[18] World Health Organization (2021). WHO Laboratory Manual for the Examination and Processing of Human Semen.

[19] Luongo F.P., Perez Casasus S., Haxhiu A., Barbarulo F., Scarcella M., Governini L., Piomboni P., Scarica C., Luddi A. (2023). Exposure to Cumulus Cell Secretome Improves Sperm Function: New Perspectives for Sperm Selection In Vitro. Cells.

[20] Sati L., Cayli S., Delpiano E., Sakkas D., Huszar G. (2014). The Pattern of Tyrosine Phosphorylation in Human Sperm in Response to Binding to Zona Pellucida or Hyaluronic Acid. Reprod. Sci..

[21] Pérez Casasús S., Luongo F.P., Haxhiu A., Orini M., Scupoli G., Governini L., Piomboni P., Buratini J., Dal Canto M., Luddi A. (2024). Paternal Age Amplifies Cryopreservation-Induced Stress in Human Spermatozoa. Cells.

[22] Festucci F., Annunzi E., Pepe M., Curcio G., D’Addario C., Adriani W. (2022). Dopamine-transporter Heterozygous Rats Carrying Maternal Wild-type Allele Are More Vulnerable to the Development of Compulsive Behavior. Synapse.

[23] Baker A.H., George S.J., Zaltsman A.B., Murphy G., Newby A.C. (1999). Inhibition of Invasion and Induction of Apoptotic Cell Death of Cancer Cell Lines by Overexpression of TIMP-3. Br. J. Cancer.

[24] Piomboni P., Gambera L., Serafini F., Campanella G., Morgante G., De Leo V. (2008). Sperm Quality Improvement after Natural Anti-Oxidant Treatment of Asthenoteratospermic Men with Leukocytospermia. Asian J. Androl..

[25] Ickowicz D., Finkelstein M., Breitbart H. (2012). Mechanism of Sperm Capacitation and the Acrosome Reaction: Role of Protein Kinases. Asian J. Androl..

[26] Donà G., Tibaldi E., Andrisani A., Ambrosini G., Sabbadin C., Pagano M.A., Brunati A.M., Armanini D., Ragazzi E., Bordin L. (2020). Human Sperm Capacitation Involves the Regulation of the Tyr-Phosphorylation Level of the Anion Exchanger 1 (AE1). Int. J. Mol. Sci..

[27] Lu Z.-J., Shi W.-J., Ma D.-D., Zhang J.-G., Long X.-B., Li S.-Y., Gao F.-Z., Zhang Q.-Q., Ying G.-G. (2024). The Azole Biocide Climbazole Induces Oxidative Stress, Inflammation, and Apoptosis in Fish Gut. Sci. Total Environ..

[28] Liao X.-L., Chen Z.-F., Zou T., Lin Z.-C., Chen X.-F., Wang Y., Qi Z., Cai Z. (2021). Chronic Exposure to Climbazole Induces Oxidative Stress and Sex Hormone Imbalance in the Testes of Male Zebrafish. Chem. Res. Toxicol..

[29] Lu Z.-J., Shi W.-J., Gao F.-Z., Ma D.-D., Zhang J.-G., Li S.-Y., Long X.-B., Zhang Q.-Q., Ying G.-G. (2023). Climbazole Causes Cell Apoptosis and Lipidosis in the Liver of Grass Carp. Aquat. Toxicol..

[30] Adamus J., Feng L., Hawkins S., Kalleberg K., Lee J. (2017). Climbazole Boosts Activity of Retinoids in Skin. Int. J. Cosmet. Sci..

[31] Pople J.E., Moore A.E., Talbot D.C.S., Barrett K.E., Jones D.A., Lim F.L. (2014). Climbazole Increases Expression of Cornified Envelope Proteins in Primary Keratinocytes. Int. J. Cosmet. Sci..

[32] Petricca S., Carnicelli V., Luzi C., Cinque B., Celenza G., Iorio R. (2023). Oxidative Stress, Cytotoxic and Inflammatory Effects of Azoles Combinatorial Mixtures in Sertoli TM4 Cells. Antioxidants.

[33] Grasa P., Colas C., Gallego M., Monteagudo L., Muiño-Blanco T., Cebrián-Pérez J.Á. (2009). Changes in Content and Localization of Proteins Phosphorylated at Tyrosine, Serine and Threonine Residues during Ram Sperm Capacitation and Acrosome Reaction. Reproduction.

[34] Yu J., Duan Y., Lu Q., Chen M., Ning F., Ye Y., Lu S., Ou D., Sha X., Gan X. (2024). Cytochrome c Oxidase IV Isoform 1 (COX4-1) Regulates the Proliferation, Migration and Invasion of Trophoblast Cells via Modulating Mitochondrial Function. Placenta.

[35] Sharma R.K., Agarwal A. (1996). Role of Reactive Oxygen Species in Male Infertility. Urology.

[36] Kim K., Kwon J.-S., Ahn C., Jeung E.-B. (2022). Endocrine-Disrupting Chemicals and Their Adverse Effects on the Endoplasmic Reticulum. Int. J. Mol. Sci..

[37] Lahimer M., Abou Diwan M., Montjean D., Cabry R., Bach V., Ajina M., Ben Ali H., Benkhalifa M., Khorsi-Cauet H. (2023). Endocrine Disrupting Chemicals and Male Fertility: From Physiological to Molecular Effects. Front. Public Health.

