# Supplementary material for: In Vitro Exposure to the Endocrine-Disrupting Chemical Climbazole Impairs Human Sperm Motility, Hormonal Signalling, and Mitochondrial Activity
Source: Cells. 2025 Mar 13;14(6):427. doi: 10.3390/cells14060427 (PMC11940937; doi:10.3390/cells14060427)
Supplement: Supplementary file 1 [file cells-14-00427-s001.zip › cells-3488397-supplementary.pdf]

Supplementary Materials

Supp. Table S1 Characteristics of primary and secondary antibodies used for immunofluorescence staining and Western Blots.

Antigen	Donor Species	Dilution	Manufacturer	RRID
**Primary antibodies**				
P-Tyrosine	Mouse	1:50-1:250	Santa Cruz Biotechnology	AB_628123
COX-4	Rabbit	1:400	Thermo Fisher	AB_10987478
B Tubulin	Mouse	1:100	Thermo Fisher	AB_2609649
**Secondary antibodies**				
Anti-Mouse-IgG HRP	Goat	1:8000	Bio-Rad laboratories	AB_609692
Anti-Rabbit-IgG HRP	Goat	1:8000	Bio-Rad laboratories	AB_1102634
Alexa Fluor Anti-Mouse 568	Goat	1:500	Thermo Fisher	AB_1500898

Supp. Table S2 List of primers used for qRT–PC

Gene	Acronymous	Chr. localization	Primer set
**Glyceraldehyde-3-Phosphate Dehydrogenase**	***GAPDH***	*chr12:6,534,512-6,538,374*	Fw 5′- ACATCGCTCAGACACCATG -3′<br>Rev 5′ – TGTAGTTGAGGTCAATGAAGGG – 3′<br>qHsaCEP0041396
**Beta-2-Microglobulin**	***B2M***	*chr15:44,711,358-44,718,851*	Fw 5′ – GGACTGGTCTTTCTATCTCTTGTAC – 3′<br>Rev 5′ – ACCTCCATGATGCTGCTTAC – 3′<br>qHsaCIP0029872
**RNA, 18S Ribosomal**	***18S***	*chr13*	Fw 5′ – GGGAGGTAGTGACGAAAAATAAC – 3′<br>Rev 5′ –TTGCCCTCCAATGGATCCT – 3′
**Androgen Receptor**	***AR***	*chrX:67,544,021-67,730,619*	Fw 5′-TTGTCCATCTTGTCGTCTTCG-3′<br>Rev 5′-ACCAAGTTTCTTCAGCTTCCG-3′
**BCL2 Apoptosis Regulator**	***BCL2***	*chr18:63,123,346-63,320,128*	Fw 5′-GGCTGGGATGCCTTTGTG -3′<br>Rev 5′-GCCAAACTGAGCAGAGTCTTCA -3′
**BCL2 Associated X**	***BAX***	*chr19:48,954,815-48,961,798*	Fw 5′-TGGAGCTGCAGAGGATGATTG -3′<br>Rev 5′-TTGCCGTCAGAAAACATGTCA -3′
**Estrogen Receptor 1**	***ESR1***	*chr6:151,656,672-152,129,619*	qHsaCEP0049903
**Estrogen Receptor 2**	***ESR2***	*chr14:64,084,232-64,338,613*	qHsaCEP0052206
**Follicle Stimulating Hormone Receptor**	***FSHR***	*chr2:48,962,157-49,154,527*	qHsaCEP0054002
**Cytochrome C Oxidase Subunit 4I1**	***COX4I1***	*chr16:85,798,633-85,807,068*	Hs00971639_m1

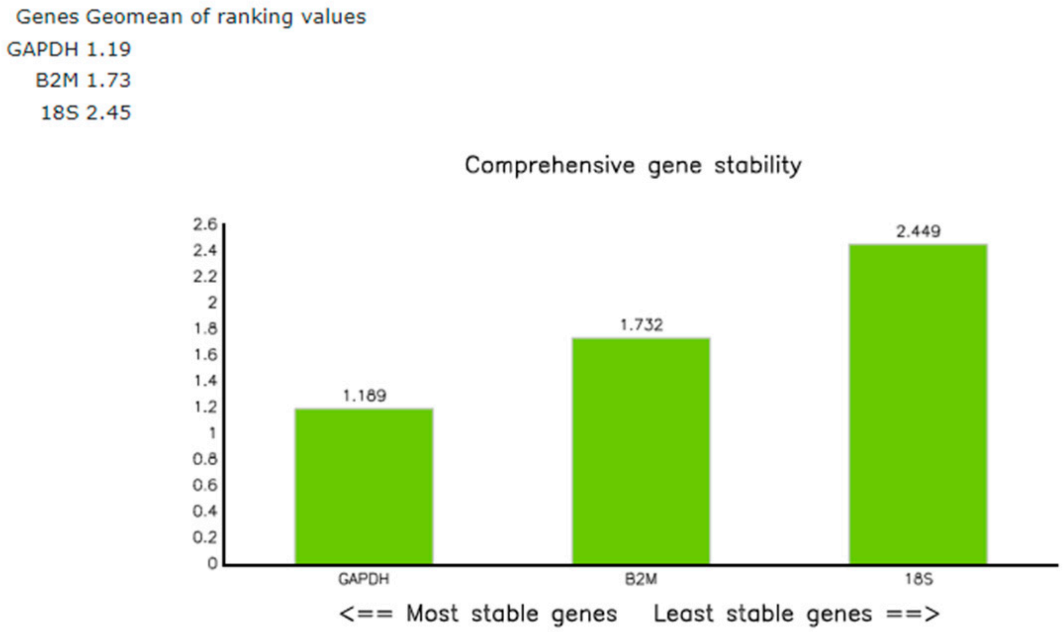

Supp. Figure S1 Gene stability analysis between housekeeping genes: *GAPDH, B2M, 18S*

Supp. Table S3 Percentage of sperm vitality in relation to time between control, DMSO 0,1% and treatment with CBZ. Significant differences are indicated with Bonferroni correction **p<0.01; ***p<0.001.

**Bonferroni's multiple comparisons test**	**Mean and SD**		**p Value**
Control vs. DMSO 0.1%	77.29±2.19	77.29±2.46	>0,9999
Control vs. 1µM	77.29±2.19	75.14±2.57	>0,9999
Control vs. 5 µM	77.29±2.19	74.71±2.21	0,9862
Control vs. 10 µM	77.29±2.19	74.29±3.24	0,4554
Control vs. 25 µM	77.29±2.19	71.57±3.47	**0,0012****
Control vs. 50 µM	77.29±2.19	70.71±3.87	**0,0001*****
DMSO 0.1% vs. 1 µM	77.29±2.46	75.14±2.57	>0,9999
DMSO 0.1% vs. 5 µM	77.29±2.46	74.71±2.21	0,9862
DMSO 0.1% vs. 10 µM	77.29±2.46	74.29±3.24	0,4554
DMSO 0.1% vs. 25 µM	77.29±2.46	71.57±3.47	**0,0012****
DMSO 0.1% vs. 50 µM	77.29±2.46	70.71±3.87	**0,0001*****

Supp. Table S4 Percentage of sperm motility in relation to time between vehicle and treatment with CBZ. Significant differences are indicated with Bonferroni correction *p<0.05; ***p<0.001.

Bonferroni's multiple comparisons test	Mean and SD		p Value
**Non-capacitation**			
Vehicle vs. 1μM	55.57±2.37	46.86±2.82	**0.0285***
Vehicle vs. 10 μM	55.57±2.37	42.86±3.57	**0.0002*****
**Capacitation**			
Vehicle vs. 1 μM	61.71±1.75	54.43±2.75	0.1299
Vehicle vs. 10 μM	61.71±1.75	49.71±2.33	**0.0005*****

Supp. Table S5 Normalized gene expression between vehicle and samples treated with CBZ calculated by Delta–Delta Ct (ΔΔCt) method. Significant differences are indicated with Bonferroni's correction *p<0.05; **p<0.01; ****p<0.0001.

Bonferroni's multiple comparisons test		p Value	
		**Non-capacitation**	**Capacitation**
ESR1	Vehicle vs. 1μM	**0.0189***	**0.0202***
	Vehicle vs. 10 μM	**0.0129***	**0.0017****
ESR2	Vehicle vs. 1μM	**0.0350***	**<0.0001******
	Vehicle vs. 10 μM	**<0.0001******	**<0.0001******
FSHR	Vehicle vs. 1μM	**<0.0001******	**<0.0001******
	Vehicle vs. 10 μM	**<0.0001******	**0.0049****
AR	Vehicle vs. 1μM	**0.0015****	**<0.0001******
	Vehicle vs. 10 μM	**0.0015****	**<0.0001******
BAX	Vehicle vs. 1μM	**0.0072****	**<0.0001******
	Vehicle vs. 10 μM	**0.0281***	**<0.0001******
BCL2	Vehicle vs. 1μM	0.6279	**<0.0001******
	Vehicle vs. 10 μM	>0.9999	**<0.0001******
COX4I1	Vehicle vs. 1μM	**<0.0001******	>0.9999
	Vehicle vs. 10 μM	**<0.0001******	**0.0024****

Supp. Table S6 Spearman's r and p-value from the correlation analysis between *ESR1, ESR2, AR,* and *FSHR* reported through the heatmap graph. *p<0.05; ***p<0.001.

**Non-capacitation**	*ESR1*	*ESR2*	*AR*	*FSHR*	
ESR1		**0.001****	**0.024***	0.239	**pValue**
ESR2	1.0		**0.024***	0.239	
AR	-0,8660	-0,8660		**0.024***	
FSHR	0,5	0,5	-0,8660		
	**Spearman r**				

**Capacitation**	*ESR1*	*ESR2*	*AR*	*FSHR*	
ESR1		0.239	**0.001*****	0.239	**pValue**
ESR2	0.500		0.239	**0.001*****	
AR	1.0	0.500		0.239	
FSHR	-0.500	-1.0	-0.500		
	**Spearman r**				
